# Marine microbial biodiversity, bioinformatics and biotechnology (M2B3) data reporting and service standards

**DOI:** 10.1186/s40793-015-0001-5

**Published:** 2015-05-08

**Authors:** Petra ten Hoopen, Stéphane Pesant, Renzo Kottmann, Anna Kopf, Mesude Bicak, Simon Claus, Klaas Deneudt, Catherine Borremans, Peter Thijsse, Stefanie Dekeyzer, Dick MA Schaap, Chris Bowler, Frank Oliver Glöckner, Guy Cochrane

**Affiliations:** 1 European Nucleotide Archive, EMBL-EBI, Wellcome Trust Genome Campus Hinxton, Cambridge CB10 1SD, UK; 2 PANGAEA - Data Publisher for Earth & Environmental Science, MARUM Center for Marine Environmental Sciences, Universität Bremen, Hochschulring 18 (Cognium), Bremen, POP 330 440, 28359, Germany; 3 Max Planck Institute for Marine Microbial Ecology, Microbial Genomics and Bioinformatics Group, Celsiusstr. 1, Bremen, 28359, Germany; 4 Oxford e-Research Centre (OeRC), University of Oxford, 7Keble Road, Oxford, UK; 5 Vlaams Instituut voor de Zee, InnovOcean site, Wandelaarkaai 7, Oostende, B-8400, Belgium; 6 IFREMER-Centre de BREST, IDM/SISMER, ZI de la Pointe du Diable, Plouzane, CS 10070, 29280, France; 7 MARIS BV, Koningin Julianalaan 345 A 2273 JJ, Voorburg, The Netherlands; 8 Environmental and Evolutionary Genomics Section, Institut de Biologie de l’Ecole Normale Supérieure (IBENS), CNRS UMR8197 Inserm U1024, Paris, 75005, France; 9 Jacobs University Bremen gGmbH, Campusring 1, Bremen, 28759, Germany

**Keywords:** Data standard, Marine, Molecular, Biodiversity, Microbial, Bioinformatics, Reporting, Interoperability

## Abstract

Contextual data collected concurrently with molecular samples are critical to the use of metagenomics in the fields of marine biodiversity, bioinformatics and biotechnology. We present here Marine Microbial Biodiversity, Bioinformatics and Biotechnology (M2B3) standards for “*Reporting”* and “*Serving”* data. The M2B3 Reporting Standard (1) describes minimal mandatory and recommended contextual information for a marine microbial sample obtained in the epipelagic zone, (2) includes meaningful information for researchers in the oceanographic, biodiversity and molecular disciplines, and (3) can easily be adopted by any marine laboratory with minimum sampling resources. The M2B3 Service Standard defines a software interface through which these data can be discovered and explored in data repositories. The M2B3 Standards were developed by the European project Micro B3, funded under 7^th^ Framework Programme “Ocean of Tomorrow”, and were first used with the Ocean Sampling Day initiative. We believe that these standards have value in broader marine science.

## Background

An immense wealth of genetic, functional and morphological diversity in marine ecosystems remains unexplored, offering the potential for substantial scientific and biotechnological discoveries. Indeed, significant interest in this area has led to large-scale initiatives, such as Tara Oceans [1], the Global Ocean Survey [2] and Malaspina [3], that target the exploration of marine biodiversity on planetary scales. While the shared goal of such initiatives is the development of an understanding of the composition and ecology of marine microbial ecosystems, each focuses on different parts of the taxonomic breadth of ocean life and only a subset of ocean ecosystems, such as epi- meso- and bathypelagic systems. Ongoing and future marine survey projects will add value to these explorations and will continue to build a powerful marine data infrastructure from which ecosystems biology and biotechnology will derive benefit. Prerequisite for the successful exploitation of acquired data are standards that enable interoperability in the data infrastructure.

Just as marine studies span many disciplines (e.g. biological, oceanographic, molecular), use of data from marine studies requires approaches that traverse the many disciplines, asking questions, for example, of species distribution, physical oceanographic parameters, molecular biology and data licensing. Each discipline has established infrastructure and best practice for the dissemination of its data, including open data repositories, reporting and data standards and discovery and analysis portals. However, there remain major barriers when data are to be used across disciplines that relate to a lack of interoperability between standards and the lack of a consistent environment for the discovery and retrieval of data.

The Marine Microbial Biodiversity, Bioinformatics, Biotechnology Project (Micro B3) [4] unites intensive oceanographic monitoring, thorough biodiversity studies and high-throughput DNA sequencing of marine genomes, metagenomes and pan-genomes. The project addresses interdisciplinary needs in marine ecosystems biology and biotechnology by considering established best practice within the disciplines and deriving practical least-change means to align practices. Recognising that it is non-trivial to influence deeply-rooted working practices established over decades, we have delivered an extensive programme of workshop-based discussions amongst representatives of the disciplines [22,23].

This effort led to the development of two standards described here. First, the *M2B3 Reporting Standard* defines and describes fields of information to be made available with marine data sets. Second, the *M2B3 Service Standard* defines and describes a software interface through which hosts of marine data, such as the public data repositories, can present their marine data holdings.

The resulting standards were used by marine sampling stations and cruises participating in the Micro B3 sampling campaign, Ocean Sampling Day (OSD) [5], a simultaneous world-scale sample and contextual data collection to investigate dynamics and functions of marine microbial diversity. We believe that our work will also be of value to other marine surveys in the future.

### M2B3 Reporting Standard

**Figure 1:**
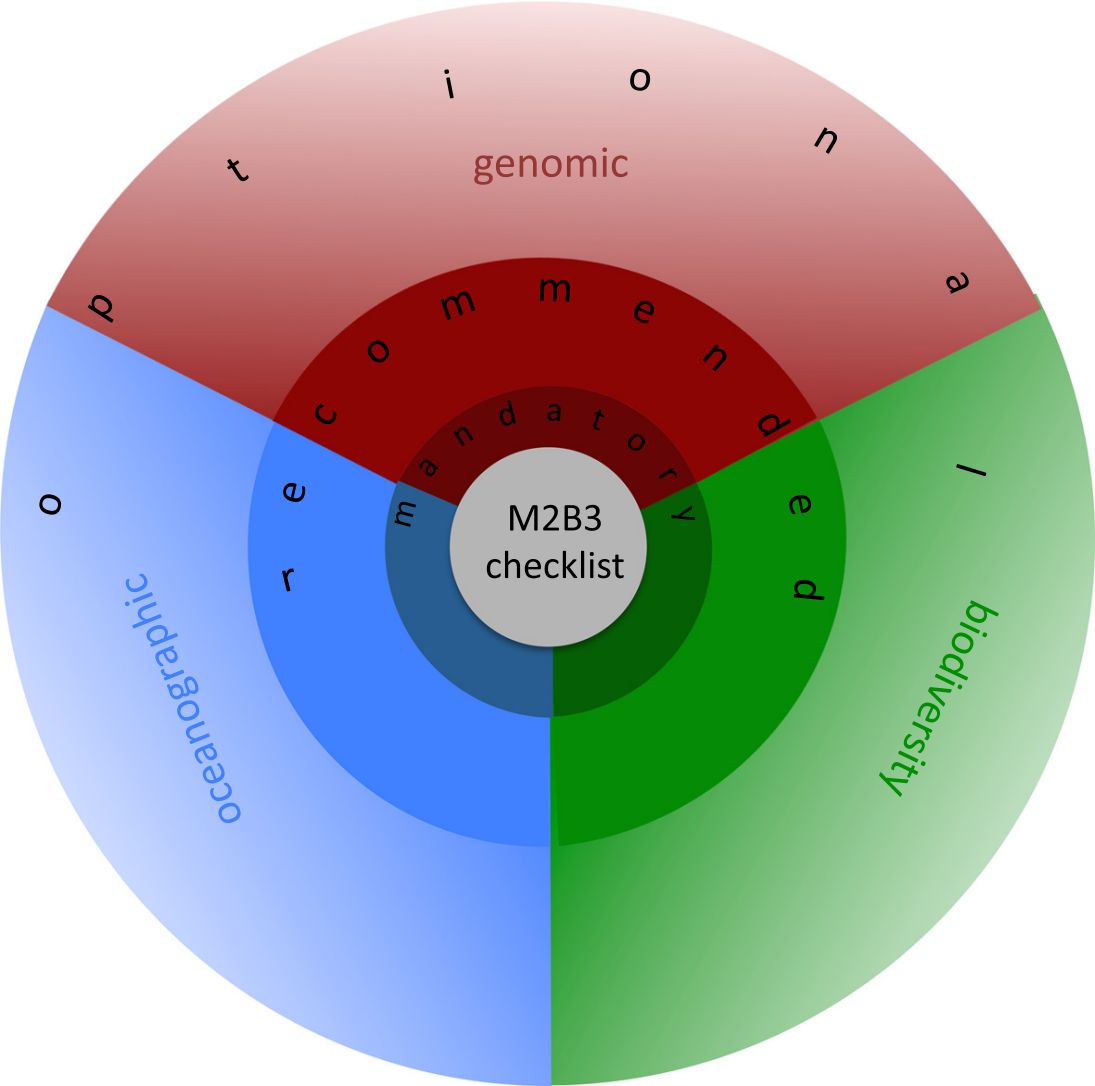
M2B3 Reporting Standard descriptors schematically depicted on the junction of three disciplines, adopting existing standards of each domain.

We have developed the M2B3 Reporting Standard to support data collection and dissemination for those involved in marine microbial sampling. The standard, shown in full in Tables 1,2,3,4,5,6,7 spans the biodiversity, molecular and oceanographic domains and adopts existing standards of each discipline with their mandatory, recommended and optional descriptors (fields of information) (see Figure 1). It represents a unique intersection of existing reporting requirements across all three domains.

**Table 1:** M2B3 Reporting Standard about an investigation effort

	**Descriptor name**	**Description of usage**	**Control vocabulary/format/unit**	**Example**
	INVESTIGATION_ **Campaign**	Refers to a sampling activity that is either determined in time, repeated in time or continuous, e.g. a cruise, a mesocosm experiment, a time series, or live data streams	Free text	Micro B3-OSD2014
	INVESTIGATION _**Site**	Refers to the unique identifier and name of the site/station where the sampling activity is performed.	Format: <Site ID from OSD Site Registry >, <Site name from OSD Site Registry>	OSD5, Poseidon-E1-M3A Time Series Station
	INVESTIGATION _**Platform**	Refers to the specific unique stage from which the sampling device was deployed; includes the platform category and platform name.	Format: <Platform category from SDN:L06>,<Platform name>	research vessel, FILIA
	INVESTIGATION _Authors	List of people who will appear in the citation of data publications. Please order the list according to authorship. The first author is the contact person.	Format: <LASTNAME>, <FirstName>, <Institution>, <email>	JONES, Peter, Institute1, pjones@institute1.eu; SMITH, Mary, Institute2, msmith@institute2.eu
	INVESTIGATION _Project	Refers to the project that organised/funded the data/sample collection.	Free text	Micro B3
	INVESTIGATION _Objective	Describes the scientific context/interest of the sampling activity. This information is useful to generate a short abstract as part of the data set citation.	Free text;	A short abstract
			100-500 words	

Mandatory information is in bold and other fields are recommended OSD Sites Registry is a controlled register for OSD sampling Sites (http://mb3is.megx.net/osd-registry). SDN:L06::XX is a controlled terms list describing “CATEGORIES” of platforms (http://seadatanet.maris2.nl/v_bodc_vocab_v2/search.asp?lib=L06).

**Table 2:** M2B3 Reporting Standard about a sampling event

	**Descriptor name**	**Description of usage**	**Control vocabulary/format/unit**	**Example**
	EVENT_**Date/Time**	Date and time when the sampling event started and ended, e.g. each CTD cast, net tow, or bucket collection is a distinct event.	Date and time in UTC;	2013-06-21T14:05:00Z/
			Format: yyyy-mm-ddThh:mm:ssZ	2013-06-21T14:46:00Z
	EVENT_**Longitude**	Longitude of the location where the sampling event started and ended, e.g. each CTD cast, net tow, or bucket collection is a distinct event.	Format: ###.######	035.666666
			Decimal degrees; East = +, West = -Format: Use WGS 84 for GPS data	035.670200
	EVENT_**Latitude**	Latitude of the location where the sampling event started and ended, e.g. each CTD cast, net tow, or bucket collection is a distinct event.	Format: ##.######	−24.666666
			Decimal degrees; North = +, South = -Format: Use WGS 84 for GPS data	-24.664300
	EVENT_Device	Refers to the instrument/gear used to collect the sample or the sensor used to measure environmental parameters.	Free text	10L-Niskins or 5L-Bucket
	EVENT_Method	Refers to the standard deployment procedure of the Device.	Free text	12 Niskins were deployed on a Rosette
	EVENT_Comment	Report any observation/deviation from the standard deployment procedure described in EVENT_Method	Free text	Lots of Jellyfish in the water

Mandatory information is in bold and other fields are recommended.

**Table 3:** M2B3 Reporting Standard about a sample

	**Descriptor name**	**Description of usage**	**Control vocabulary/format/unit**	**Example**
	SAMPLE_**Title**	A short informative description of the sample. Must be unique for each sample, (i.e. for each filter generated during sampling).	Format: <OSD_SiteID > _ < Month > _ < Year > _ < SiteName > _ < Protocol_Label > _ < SampleNo > _ < Depth>	OSD3_06_14_Helgoland_NPL022_1_surface
	SAMPLE_**Depth**	The distance below the surface of the water at which a measurement was made or a sample was collected.	Format: ##.#;	1.5
			Positive below the sea surface.	
			SDN:P06:46:ULAA for m	
	SAMPLE_**Protocol_Label**	Identifies the protocol used to produce the sample, e.g. filtration and preservation.	Term list;	NPL022
			See the *SAMPLE_Protocol_Label* in the OSD Protocols Section for details	
	SAMPLE_Quantity	Refers to the quantity of environment that was sampled, most often with dimensions Length, Amount, Mass or Time.	Format : ###.###	20 Litres
			See the *SAMPLE_Quantity* in the OSD Protocols Section for details	
	SAMPLE_Container	Refers to the container in which the sample is stored prior to analysis.	Term list;	Sterivex cartridge
			See the *SAMPLE_Container* in the OSD Protocols Section for details	
	SAMPLE_Content	Refers to the content of the sample container. While the sample might target a specific organism (e.g. bacteria), the sample content might be a filter or a volume of water.	Term list;	Particulate matter on a 0.22 μm pore size filter
			See the *SAMPLE_Content* in the OSD Protocols Section for details	
	SAMPLE_Size-Fraction_Upper-Threshold	Refers to the mesh/pore size used to pre-filter/pre-sort the sample. Materials larger than the size threshold are excluded from the sample.	Term list;	no pre-filtration
			See the *SAMPLE_Size-Fraction_Upper-Threshold* in the OSD Protocols Section for details; in μm	
	SAMPLE_Size-Fraction_Lower-Threshold	Refers to the mesh/pore size used to retain the sample. Materials smaller than the size threshold are exclude from the sample.	Term list;	0.22
			See the *SAMPLE_Size-Fraction_Lower-Threshold* in the OSD Protocols Section for details; in μm	
	SAMPLE_Treatment_Chemicals	Refers to the chemicals (e.g. preservatives) added to the sample.	Terms list: ChEBI;	None
			See the *SAMPLE_Treatment_Chemicals* in the OSD Protocols Section for details	
	SAMPLE_Treatment_Storage	Refers to the conditions in which the sample is stored, e.g. temperature, light conditions, time.	Term list;	−80 degrees Celsius
			See the *SAMPLE_Treatment_Storage* in the OSD Protocols Section for details	

Mandatory information is in bold and other fields are recommended. OSD Protocols are available at http://www.microb3.eu/sites/default/files/osd/OSD_Handbook_v2.0.pdf. ChEBI is an ontological classification and dictionary of small chemical compounds (http://www.ebi.ac.uk/chebi/init.do).

**Table 4:** M2B3 Reporting Standard about the sample environmental context

**Descriptor name**	**Description of usage**	**Control vocabulary/format/unit**	**Example**
**ENVIRONMENT_Biome**	Descriptor of the broad ecological context of a sample.	Terms list: EnvO	ENVO:01000023 for “marine pelagic biome”
ENVIRONMENT_**Feature**	Compared to biome, feature is a descriptor of a geographic aspect or a physical entity that strongly influences the more local environment of a sample.	Terms list: EnvO	ENVO:00000209 for “photic zone”
ENVIRONMENT_**Material**	Descriptor of the material that was displaced by the sampling activity, or material in which a sample was embedded, prior to the sampling event.	Terms list: EnvO	ENVO:00002149 for “sea water”
ENVIRONMENT_**Temperature**	Temperature of water at the time of taking the sample. Define the parameter according to Table 7.	Format: ##.#	16.2°C
		SDN:P02:75:TEMP	
		SDN:P06:46:UPAA for°C	
ENVIRONMENT_**Salinity**	Salinity of water at the time of taking the sample. Define the measurement according to Table 7.	Format: ##.#	39.1 psu
		SDN:P02:75:PSAL	
		SDN:P06:46:UGKG for PSU	
ENVIRONMENT_Marine_Region	It characterises the environment, based on the latitude and longitude, by reference to geographic, political, economic or ecological boundaries.	Terms list: Marine Regions	MRGID:21886 for Marine Ecoregion:South European Atlantic Shelf
ENVIRONMENT_Other_Parameters	Add as many fields as there are other environments parameters measured.		
	Define the measurement according to Table 7.		
	See the list of recommended environmental parameters in Table 5		

Mandatory information is in bold and other fields are recommended EnvO is the Environment Ontology (http://www.environmentontology.org/Browse-EnvO). SDN:P02:75:XXXX is a controlled terms list describing “WHAT” is measured (http://seadatanet.maris2.nl/v_bodc_vocab_v2/search.asp?lib=P02). SDN:P06:46:XXXX is a controlled terms list describing “UNITS” of measurements (http://seadatanet.maris2.nl/v_bodc_vocab_v2/search.asp?lib=P06). Marine Regions is a standard list of marine georeferenced place names (http://www.marineregions.org/).

**Table 5:** M2B3 Reporting Standard about environmental measurements

**Interest**		**Measurement**	**Description of usage**	**Control vocabulary/format/unit**
	General	Conductivity	Electrical conductivity of water	SDN:P02:75:CNDC
				SDN:P06:46:UECA for mS/cm
		**Temperature**	Temperature of water	SDN:P02:75:TEMP
				SDN:P06:46:UPAA for °C
		Depth (m)	Vertical spatial coordinates	SDN:P02:75:AHGT
				SDN:P06:46:ULAA for m
		**Salinity**	Salinity of water	SDN:P02:75:PSAL
				SDN:P06:46:UGKG for PSU
		Fluorescence	Raw (volts) or converted (mg Chla/m^3) fluorescence of the water	SDN:P02:75:FVLT
				SDN:P06:46:UVLT for volts
	Nutrient status of a system	Nitrate	Nitrate concentration parameters in the water column	SDN:P02:75:NTRA
				SDN:P06:46:UPOX for μmol/L
		Nitrite	Nitrite concentration parameters in the water column	SDN:P02:75:NTRI
				SDN:P06:46:UPOX for μmol/L
		Phosphate	Phosphate concentration parameters in the water column	SDN:P02:75:PHOS
				SDN:P06:46:UPOX for μmol/L
		Silicate	Silicate concentration parameters in the water column	SDN:P02:75:SLCA
				SDN:P06:46:UPOX for μmol/L
		Ammonium	Ammonium concentration parameters in the water column	SDN:P02:75:AMON
				SDN:P06:46:UPOX for μmol/L
	Chemical properties of a system	pH	Alkalinity, acidity and pH of the water column	SDN:P02:75:ALKY
		Dissolved oxygen concentration	Dissolved oxygen parameters in the water column	SDN:P02:75:DOXY
				SDN:P06:46:KGUM for μmol/kg
	Optical properties of a system	Downward PAR	Visible waveband radiance and irradiance measurements in the water column	SDN:P02:75:VSRW
				SDN:P06:46:UMES for μE/m^2/s
		Turbidity	Transmittance and attenuance of the water column	SDN:P02:75:ATTN
				SDN:P06:46:USTU for FTU or NTU
	Biogeochemistry (Amount or Mass)	Carbon organic particulate (POC)	Particulate organic carbon concentration in the water column	SDN:P02:75:CORG
				SDN:P06:46:UGPL for μg/L
		Nitrogen organic particulate (PON)	Particulate organic nitrogen concentration in the water column	SDN:P02:75:NTOT
				SDN:P06:46:UGPL for μg/L
		Carbon organic dissolved (DOC)	Dissolved organic carbon concentration in the water column	SDN:P02:75:DOCC
				SDN:P06:46:UPOX for μmol/L
		Nitrogen organic dissolved (DON)	Dissolved organic nitrogen concentration in the water column	SDN:P02:75:TDNT
				SDN:P06:46:UMGL for mg/L
	Ecosystem trophic structure & biodiversity (Amount, Volume or Mass of organisms in the environment)	Pigment concentrations	Concentration of pigments (e.g. chlorophyll a) extracted and analysed by fluorometry or HPLC	SDN:P02:75:CPWC
				SDN:P06:46:UMMC for mg/m^3
		Picoplankton (Flow Cytometry)	Abundance of cells in the water column (+other avail. cell properties)	SDN:P02:75:BATX
				SDN:P06:46:UPMM for #/m^3
		Nano/Microplankton	Abundance of cells in the water column (+other avail. cell properties)	SDN:P02:75:MATX or PATX
				SDN:P06:46:UPMM for #/m^3
		Meso/Macroplankton	Abundance of individuals in the water column (+other avail. properties)	SDN:P02:75:ZATX
				SDN:P06:46:UPMM for #/m^3
	Ecosystem trophic rates	Primary Production (isotope uptake)	Primary Production in the water column	SDN:P02:75:PPRD
				SDN:P06:46:UGDC for mg/m^3/d
		Primary Production (oxygen)	Primary Production in the water column	SDN:P02:75:PPRD
				SDN:P06:46:UGDC for mg/m^3/d
		Bacterial production (isotope uptake)	Bacterial production in the water column	SDN:P02:75:UPTH
				SDN:P06:46:UGDC for mg/m^3/d
		Bacterial production (respiration)	Bacterial production in the water column	SDN:P02:75:UPTH
				SDN:P06:46:UGDC for mg/m^3/d

Mandatory information is in bold and other fields are recommended.

**Table 6:** M2B3 Reporting Standard about organisms in a sample

	**Descriptor name**	**Description of usage**	**Control vocabulary/format/unit**	**Example**
	ORGANISM_**Taxon_ID**	An identifier for the nomenclatural (not taxonomic) details of a scientific name.	Terms list: WoRMS	urn:lsid:marinespecies.org:taxname: 345516
			Format: LSID	
	ORGANISM_**Taxon_Scientific_Name**	The full name of the lowest level taxon.	Terms list: WoRMS	Prochlorococcus marinus
			Format: Taxon name	
	ORGANISM_ Sex	The sex of a specimen or collected/observed individual(s).	Terms list: M = Male; F = Female; H = Hermaphrodite; I = Indeterminate (examined but could not be determined; U = Unkown (not examined); T = Transitional (between sexes; useful for sequential hermaphrodites); B = Both Male and Female	M
	ORGANISM_Life_Stage	Indicates the life stage present.	Free text	resting spores
	ORGANISM_Size	Refers to size measurements that are made concurrently to the enumeration and identification of organisms.		
		Define the measurement according to Table 7.		
	ORGANISM_Biovolume	Refers to volume measurements/calculations that are made concurrently to the enumeration and identification of organisms.		
		Define the measurement according to Table 7.		
	ORGANISM_Biomass	Refers to biomass measurements/calculations that are made concurrently to the enumeration and identification of organisms.		
		Define the measurement according to Table 7.		

Mandatory information is in bold and other fields are recommended WoRMS is the World Register of Marine Species (http://www.marinespecies.org/aphia.php?p=search).

**Table 7:** M2B3 Reporting Standard about environmental measurement processes

	**Descriptor name**	**Description of usage**	**Control vocabulary/format/unit**	**Example**
	MEASUREMENT_**ID**	Unique ID from a controlled vocabulary.	SDN:P02:75:xxxx	SDN:P02:75:CORG for Particulate organic carbon concentration in the water column
	MEASUREMENT _Name	Common name for the measurement.	Free text	POC
	MEASUREMENT _Quantity	Describes the quantity measured using terms from the Système International of units.	Free text; SI of units	Mass concentration
	MEASUREMENT _Dimensions	Describes the quantity measured using dimension terms from the Système International of units.	Free text; SI of units	M^1 L^-3
	MEASUREMENT _Currency	May often refer to a TAXONOMY_ID or a CHEMICAL_ID.	Free text;	Organic carbon
			Terms list: WoRMS;	
			Terms list: ChEBI	
	MEASUREMENT _Units	Describes the units of the quantity measured using terms from the Système International of units.	SDN:P06:46:xxxx	SDN:P06:46:UGPL for μg/L
	MEASUREMENT _Method	Describes the measurement method used. Equivalent to methodological details provided in a paper.	Free text	Mass spectrometry
	MEASUREMENT _Comment	Any comment about the measurement.	Free text	Inorganic carbon removed by acidification

Mandatory information is in bold and other fields are recommended.

We have been strongly guided in this work by the existing standards MEDIN [6], MIxS [7] and Darwin Core [8], the expertise of the Tara Oceans project teams and the International Census of Marine Microbes (ICoMM) project [9], and knowledge of community-established reporting practice into public data archives bestowed by experts from the biodiversity, oceanographic and molecular domain.

**Figure 2:**
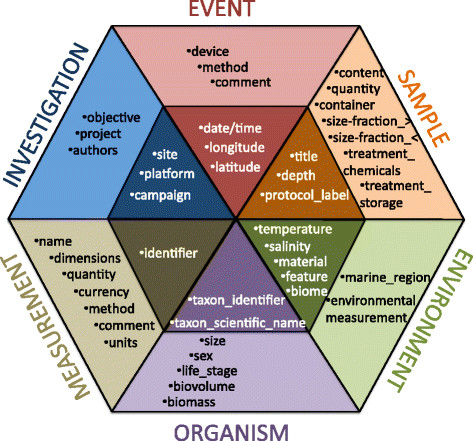
**Mandatory and recommended information of the M2B3 Reporting Standard; descriptors are split into six categories represented by coloured triangles, where mandatory descriptors are in the dark-shaded area and recommended information elements are in the light-shaded area.** Environmental measurements in the ENVIRONMENT section are further specified in Figure 3.

The core of the M2B3 Reporting Standard is the M2B3 checklist, (see Figure 2). This core represents the minimal mandatory reporting requirement and consists of descriptors essential to oceanographic, biodiversity and molecular domains, representing research on microbial diversity and function in the marine environment. Marine scientists should be able to report this minimum contextual information about each marine microbial sample irrespective of their scientific expertise and resources available for the sampling.

**Figure 3:**
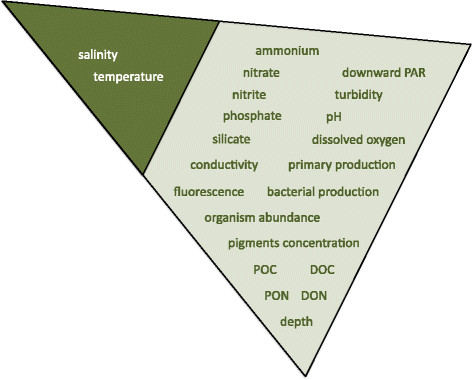
Mandatory (in the dark green area) and recommended (in the light green area) environmental measurements of the M2B3 Reporting Standard.

The M2B3 Reporting Standard includes a set of recommended descriptors (see Figure 2), provision of which brings each marine microbial sample into a rich environmental context and allows better ecological interpretation and experimental reproducibility. The standard’s environmental parameters are recommended by the Micro B3 Consortium for description of the environmental landscape of each epipelagic microbial sample (see Figure 3). Here, we have taken an approach including descriptors that draw a balance between analysis requirements-driven methods and current reporting practice in marine microbial sampling. In the requirements-driven approach we analysed several use cases from the area of diatom biology and marine prokaryotic biodiversity. Collated environmental parameters, recorded and reported in these studies in order to answer the scientific questions posed in the studies, represent the optimal list of environmental variables to be measured at the time of microbial sample collection from the epipelagic zone. The current sampling practice-driven approach is the pragmatic counterpart, where environmental variables were identified based on current marine sampling practice surveys and consultations with experts from European marine stations with established long-term sea monitoring programs and a wealth of expertise, such as Western Channel Observatory in the UK, Station Biologique de Roscoff in France, the Stazione Zoologica in Naples, Italy, or the Biological Institute Helgoland (BAH) of the Alfred Wegener Institute, the Helmholtz Centre for Polar and Marine Research in Germany.

**Figure 4:**
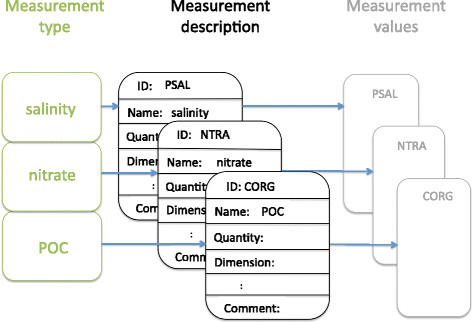
**The logical connection between environmental measurements (Table**5**), recording of the measurements (Table**7**) and measured values, shown on the example of three environmental parameters – salinity, nitrate and carbon organic particulate (POC).**

All mandatory and recommended information is described in detail in Tables 1,2,3,4,5,6,7 including specification relating to usage, formal requirements for structure, indication of appropriate units, where applicable, and an example. Descriptors are split for easy navigation into six categories: (1) the marine investigation effort, (2) the sample-taking event, (3) sample-specific details, (4a) the environmental context of the sample, (4b) environmental measurements, (5) marine species found in the sample and (6) description of environmental measurement processes. Descriptors of each conceptual category are prefixed with the category name. Table 4 specifies a broad and local environmental context of a sample including required minimum of measured environmental parameters. Table 5 focuses on specific environmental parameters that complement the fields in Table 4. Table 7 defines how environmental measurements are captured. The logical relationship between the environmental measurement, measurement description and measurement values is summarised in Figure 4.

### M2B3 Reporting standard compliance

It is worth noting that if all mandatory descriptors from the M2B3 Reporting Standard are reported by a sampling station or a cruise, then a data management centre is frequently able to infer additional descriptors available from the public record. In one example, relating to OSD, the Micro B3 Information System (Micro B3 IS) [10] and the OSD coordinators are able to infer additional descriptors available from public data archives, such as the Data Publisher for Earth and Environmental Science (PANGAEA) for environmental data [11],[12] and the European Nucleotide Archive (ENA) for molecular data [13],[14]. The additional information can be added *post hoc* for all samples acquired within the OSD campaign since the campaign has standardised and published sampling protocols and a Registry of OSD stations and cruises [15]. The inferred descriptors include, for instance, a sample catalogue number and collection code assigned by the bio-archiving institution where the OSD samples will be centrally deposited. In a second example that applies very broadly across marine samples, remotely sensed data (such as cloud cover, air temperature and wind conditions) can be connected to appropriate records based upon geospatial fields.

Combining information compliant with the M2B3 Reporting Standard from marine sampling laboratories with inferred information has two major advantages: (1) it significantly reduces the reporting burden for the marine sampling laboratories and (2) it ensures that OSD data records created at the molecular data archive will be compliant with the MIxS molecular data standard, Version 4 [16], OSD data records created at the oceanographic data archive will be compliant with the oceanographic Common Data Index (CDI), Version 3 [17] and OSD data records created at the biodiversity data archive will be compliant with the biodiversity OBIS Schema, Version 1.1 [18].

### M2B3 service standard

Six descriptors from the M2B3 Reporting Standard are central to data interoperability across disciplines. These descriptors provide the basis for connecting data points from one discipline to data points in another and are thus the indices upon which data resources providing services must present their data. The interoperability descriptors are: INVESTIGATION_Site, INVESTIGATION_Platform, EVENT_Date/Time, EVENT_Longitude, EVENT_Latitude and SAMPLE_Depth.

In order for users of marine data to discover and access data, there is a need for these fields of information to be made searchable in a single and consistent way across relevant data resources.

We define the M2B3 Service Standard as a standardised set of informatics methods through which marine data can be discovered in data resources. The six interoperability descriptors are presented by a compliant data resource using a programmatic service interface that follows Open Geospatial Consortium (OGC) standards, the Web Map Service (WMS), Web Feature Service (WFS) and/or the OpenSearch protocol. To date, the European Nucleotide Archive, European Ocean Biogeographic Information System (EurOBIS) [19],[20], Micro B3 Information System, PANGAEA and SeaDataNet [21] have committed to supporting the M2B3 Service Standard for OSD data.

## Conclusions

The M2B3 Reporting Standard combines reporting requirements of three disciplines. Compliance with the standard ensures that the collected data can be correctly directed to and stored in their respective domain-specific data archives, which are the ENA for molecular data and PANGAEA for environmental data and morphology-based biodiversity data. Compliance with the standard allows PANGAEA to create a condensed metadata summary and share it with pan-European oceanographic and biodiversity information networks, managed by SeaDataNet and EurOBIS, respectively. Micro B3 IS and other data resources compliant with the M2B3 Service standard can discover marine data compliant with the M2B3 Reporting Standard.

During its preparation, development of the M2B3 Reporting Standard and the M2B3 Service Standard allowed experts from the oceanographic, biodiversity and molecular disciplines to review current working practice, to extract and formulate what is essential and universal and to find common ground. Adoption of the M2B3 Reporting Standard will require a similar effort from the marine science community, as already started with the OSD sampling marine laboratories. The ultimate reward will be a unique collection of standardised marine data for the exploration of ecosystem biology and the advance of biotechnology.

## Abbreviations

CDI: Common Data Index

ENA: European Nucleotide Archive

EurOBIS: European Ocean Biogeographic Information System

ICoMM: International Census of Marine Microbes

M2B3: Marine Microbial Biodiversity, Bioinformatics and Biotechnology

Micro B3: Marine Microbial Biodiversity, Bioinformatics, Biotechnology

Micro B3 IS: Micro B3 Information System

OGC: Open Geospatial Consortium

OSD: Ocean Sampling Day

PANGAEA: Data Publisher for Earth and Environmental Science

SDN: SeaDataNet

WFS: Web Feature Service

WMS: Web Map Service; UTC: Coordinated Universal Time; WGS84: World Geodetic System 1984; GPS: Global Positioning System; LSID: Life Science Identifier.

## Competing interests

The authors declare that they have no competing interests.

## Authors’ contributions

PH coordinated the M2B3 standards development; SP contributed to the marine aspect of the M2B3 reporting standard, RK, GC, PH, MB and AK contributed to its molecular aspect and SC, KD CB and SD contributed to its biodiversity aspect; G.C DS, PT, RK and SC were leading the work on the M2B3 service standard. CB advised on the use case studies, GC and FOG provided overall guidance. PH wrote the manuscript with an editorial contribution of SP and GC and a revision by all co-authors. All authors read and approved the final manuscript.
